# Community-centered approaches to aquaculture in small-scale fisheries

**DOI:** 10.1007/s13280-025-02302-w

**Published:** 2025-12-18

**Authors:** Liliana Sierra Castillo, Christine Knott, Anastasia C. E. Quintana, Ana K. Spalding, Erendira Aceves-Bueno, Jessica Blythe, Antonella Rivera, Bonnie Basnett

**Affiliations:** 1 Bren School of Environmental Sciences and Management, University of California, Santa Barbara, CA USA; 2 Ocean Nexus, Marine Affairs Program, University of Rhode Island, Kingston, USA; 3 Smithsonian Tropical Research Institute, Panama City, Panama; 4 Department of Women’s Studies, Arts and Letters, San Diego State University, 5500 Campanile Dr, San Diego, CA 92182 USA; 5 School of Marine and Environmental Affairs, University of Washington, Seattle, USA; 6 Environmental Sustainability Research Centre, Brock University, St. Catharines, ON Canada; 7 Coral Reef Alliance, Coral Triangle, Honduras

**Keywords:** Blue economy, Blue transformation, Coastal communities, Small-scale aquaculture, Small-scale fisheries

## Abstract

Aquaculture is the fastest-growing seafood production system globally, offering economic and social opportunities for small-scale fishing communities. Yet, it is often introduced through top-down, technocratic approaches that ignore the social-ecological realities of these communities. Drawing on four case studies from Mexico and Honduras, this study uses a multistage, participatory mixed-methods approach to examine the integration of aquaculture into existing fisheries. We focus on governance, social organization, economic assets, and cultural traditions, key yet understudied dimensions of implementation. Findings reveal that projects overlooking these interactions risk deepening inequities, displacing traditional livelihoods, and weakening community cohesion. Success depends on early economic support, recognition of local traditions and social structures, and the creation of context-specific governance systems. Centering community needs and experiences can help design equitable, place-based aquaculture initiatives that strengthen, rather than disrupt, small-scale fishing livelihoods.

**Supplementary Information:**

The online version contains supplementary material available at 10.1007/s13280-025-02302-w.

## Introduction

Since the 1980s, global aquaculture production has expanded rapidly (Cottrell et al. 2021; Costello et al. 2023; Froehlich et al. 2023), and for the first time in 2022, it surpassed wild-caught fisheries in output (Froehlich et al. 2023; FAO Fisheries and Aquaculture Dept, 2024; Garlock et al. 2024). Aquaculture development has attracted significant attention from various stakeholders, including government agencies, non-governmental organizations, and development organizations, due to its potential to provide nutrition, economic and ecological gains, and social benefits, particularly to vulnerable communities, and it is a key component of development narratives such as the Blue Economy (Fröcklin et al. 2012; Krause et al. 2020; Farmery et al. 2021; Brugere et al. 2023).

Yet realizing these promises depends heavily on how aquaculture is implemented and the extent to which local perspectives are integrated into its development. Understanding aquaculture implementation from community perspectives is essential, as it can leverage existing capacities to maximize economic gains while ensuring social benefits and reducing trade-offs (D’Anna and Murray 2015; Mansfield et al. 2024). When locally relevant considerations are integrated, aquaculture has the potential to improve food security and economic stability (Belton and Thilsted 2014; Fong et al. 2024; Short et al. 2021), diversify livelihoods, and minimize ecological risks such as the escape of non-native species (Garlock et al. 2024; Sumaila et al. 2022). It can also mitigate social challenges, including the displacement of traditional fishers, disruptions to local markets and trade (Campbell et al. 2021; Damonte et al. 2023), and changes in cultural practices. However, in practice, the transition from fishing to aquaculture is often implemented in a haphazard manner that overlooks the unique social, cultural, economic, and ecological characteristics of existing fishery systems, particularly for small-scale fisheries in the Global South (Cottrell et al. 2021; D’Anna and Murray 2015; Nahuelhual et al. 2019; Mansfield et al. 2024).

For example, in Madagascar, the introduction of mariculture into small-scale fishery communities generated social conflict (Baker-Médard and Kroger 2023); in Zanzibar and Tanzania, an emphasis on production and economic gains negatively affected women’s health and well-being (Fröcklin et al. 2012); and in Chile, Indigenous fishing communities experienced displacement due to aquaculture policies that prioritized economic growth over cultural and subsistence practices (Pitchon 2015). Recent evidence suggests that integrating aquaculture alongside existing fisheries, rather than replacing them entirely, provides more favorable outcomes for these communities, as it better reflects the multifaceted values and priorities of small-scale fishery systems (Pitchon 2015; Stoll et al. 2019; Campbell et al. 2021; Farmery et al. 2021).

Moreover, aquaculture projects in fishing communities are still predominantly implemented using approaches where decision-making is driven by political and corporate interests that prioritize economic and technical outcomes in a top-down approach (Belton and Thilsted 2014; Belton et al. 2018; Krause et al. 2020; Brugere et al. 2021, 2023). These interventions often focus narrowly on technical, production, economic, and biological aspects of aquaculture, overlooking the broader complexities of the system, such as the sociocultural local context of these communities and the potential impacts of aquaculture on their way of life and well-being (Costa-Pierce 2010; Brugere et al. 2021, 2023; Mansfield et al. 2024). As a result, these initiatives frequently fail to create sustainable, equitable, long-term solutions that enhance the overall well-being of the communities (Nahuelhual et al. 2019; Krause et al. 2020; Blythe et al. 2021; Brugere et al. 2021; Roscher et al. 2022; Gerhardinger et al. 2023; Mansfield et al. 2024). They also tend to neglect critical questions, such as “aquaculture by whom and for whom,” often failing to place communities at the center of the decision-making process (Campbell et al. 2021; D’Anna and Murray 2015).

The complexity of small-scale fisheries as social–ecological systems, in which human and environmental domains are linked through dynamic feedback loops, requires integrated approaches that are often outside the scope or capacity of actors engaging in aquaculture development, such as governments and NGOs (Basurto and Ostrom 2009; Armitage et al. 2012; Armitage and Marschke 2013). As a result, top-down approaches often fail to reflect community needs and lived experiences (Brugere et al. 2023; Mansfield et al. 2024). Moreover, there is a lack of practical guidance for designing participatory strategies that foster meaningful, sustained engagement (Nagel et al. 2024). Although social–ecological frameworks have been used to explore interactions between aquaculture and small-scale fisheries (Mansfield et al. 2024), empirical case studies grounded in real-world contexts remain limited.

This study examines community experiences with small-scale aquaculture implementation in coastal fishing communities, focusing on the key factors that communities themselves identify as critical for successful integration. Addressing a key empirical gap, we explore how aquaculture and small-scale fisheries interact from the perspective of communities in Mexico and Honduras, using a multistage mixed-methods approach to capture diverse insights. Our goal is to identify shared challenges and opportunities that shape aquaculture adoption based on lived experience and local knowledge. While aquaculture development often remains top-down due to high capital requirements, understanding the social and contextual factors that matter to communities can help shift policy away from narrow technocratic models toward more inclusive, community-centered strategies. We aim for this research to inform governments and NGOs to better align implementation with local capacities. In doing so, we also offer participatory methods and guidelines that invite stakeholder engagement through inclusive and emancipatory research approaches.

## Materials and Methods

**Fig. 1:**
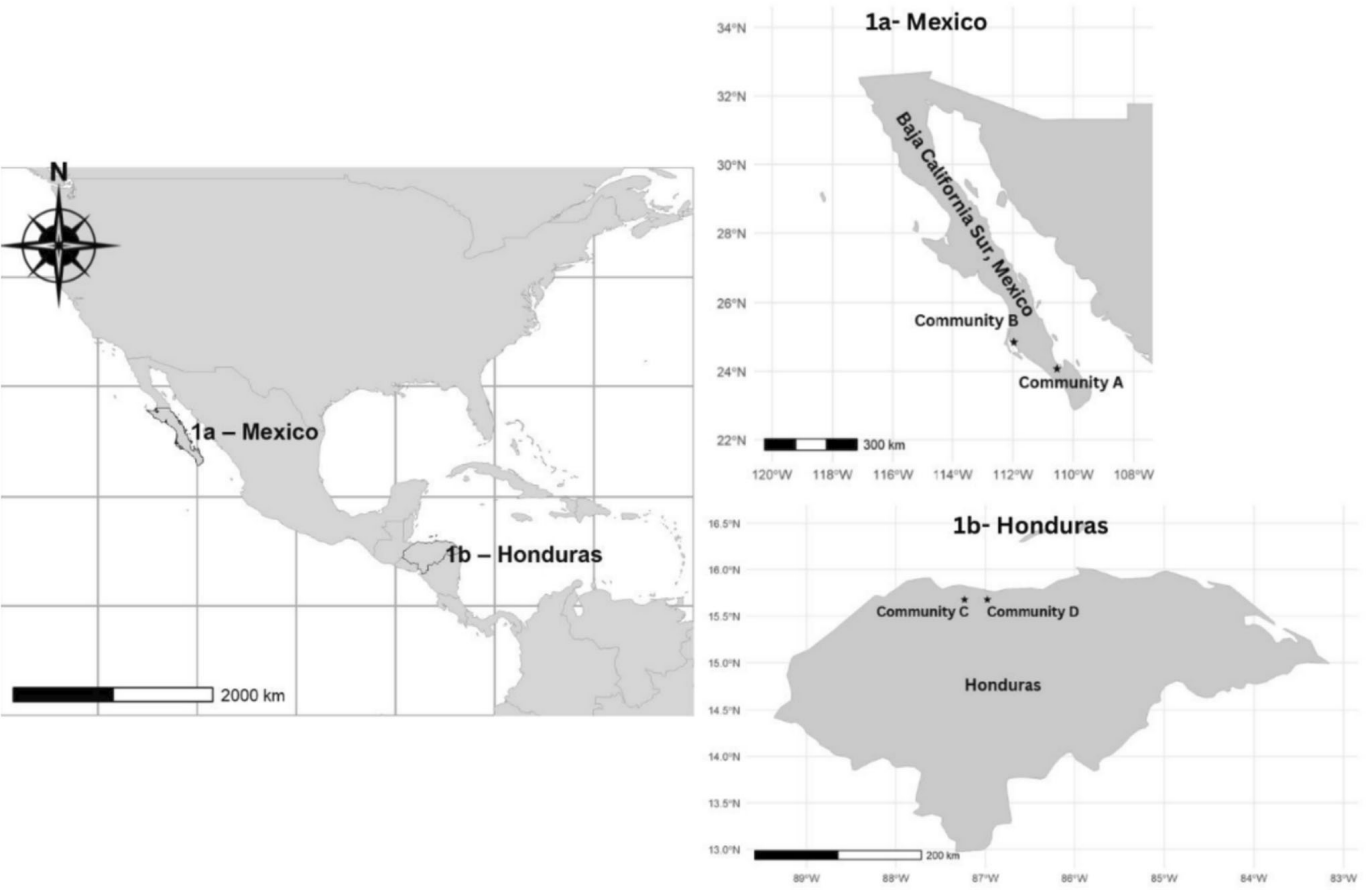
Location of communities in Mexico (**a**) and Honduras (**b**). The locations of the communities are shown with stars

This research was conducted using comparative case studies (do Amaral 2022) following a multistage mixed methods (Fig. S1) (Yawson and Greiman 2016; Janssens De Bisthoven et al. 2020; Dencer-Brown et al. 2022) following two parts: Part 1 in Mexico (Fig. 1) and Part 2 (Fig. 1) in Mexico and Honduras (Lawrence and Tar 2013; Walsh 2015). Part 1 examined the key considerations that small-scale fishing communities in Mexico view as essential for aquaculture, reflecting their lived experiences, cultural values, resources, and social dynamics.

Part 2 focused on evaluating the identified considerations from the perspective of local communities, specifically assessing their relevance for aquaculture implementation or expansion and how they interact with existing fisheries. Part 2 of the study, particularly for Honduras, incorporated a participatory research dimension, where collaboration with an NGO and local stakeholders in Honduras enabled the co-development of research questions, validation of findings, and shared reflection around aquaculture implementation. This participatory engagement complemented the broader mixed-methods design by centering community knowledge and priorities within the research process.

To capture a broad range of insights, we selected communities with varying levels of experience in aquaculture; in Mexico, communities with established aquaculture practices were included, and in Honduras, communities were in the early planning stages of aquaculture development. For this study, we will refer to the community as the group of leaders and individuals we worked with, based on their shared values, lived experiences working with aquaculture and small-scale fisheries, and ongoing collaboration. While the term “community” can be interpreted in many ways, here it aligns with literature that defines communities not solely by geography, but by social connection, mutual engagement, and a sense of shared purpose (Edwards 2011; Cobigo et al. 2016). We aimed to capture perspectives on aquaculture across diverse contexts, using the selected cases to examine how cross-sectional knowledge, particularly the transfer of lessons from more experienced communities, can inform aquaculture development in new settings. For this study, cross-sectional knowledge was interpreted as the reciprocal exchange of experiences across communities in different countries, enabling the identification of shared challenges and the generation of context-specific insights to support more grounded policy and implementation strategies (Mitton et al. 2007).

### Study sites

#### Mexico—Community A

Community A (Fig. 1) is a generational mestizo diving community in the municipality of La Paz, Baja California Sur, where fishing practices have been passed down through generations and remain central to livelihoods, cultural identity, and collective purposes. Situated next to a coastal lagoon surrounded by mangroves, the community relies on the area’s rich diversity of bivalves, including penshell, clams, and conchs, with penshell and Catarina clam being the most economically important species. In 2017, members of the community organized a fishing cooperative (~ 100 members) and obtained a lagoon concession, granting them exclusive fishing rights (SAGARPA 2017).

The community’s first aquaculture initiative began in 2013, supported by a local non-governmental organization (NGO), and focused on restoring Catarina clam populations by cultivating clam seed. Although intended to replenish wild stocks and provide economic alternatives to fishing, the project failed due to illegal fishing in the aquaculture zone and a lack of continued funding (Castillo et al. 2025). A second initiative in 2019, also NGO-funded, aimed to restore penshell populations using aquaculture as a strategy to shift community labor away from wild harvest. This effort similarly failed, due to both persistent illegal fishing and an outbreak of tunicates that affected penshell stocks (Moreno-Dávila et al. 2021). Since 2020, a new project has promoted oyster aquaculture as an alternative livelihood. This initiative also seeks to reduce fishing pressure by encouraging fisher participation in oyster cultivation, facilitating a transition toward aquaculture-based livelihoods. As of 2024, some community members remain engaged in oyster production.

#### Mexico—Community B

Community B (Fig. 1) is composed of generational fishers and farmers from a mestizo background. It is approximately 40 min by car near a coastal lagoon channel that is part of Bahia Magdalena, Baja California Sur, Mexico. This area is very important for fishing in Baja California Sur because it is the most extensive wetland ecosystem of the area, resulting in high volumes of diverse fish and bivalves (Ojeda-Ruiz et al. 2018, 2019). The main fishing methods used are hook and line, nets, and diving, catching multiple species such as shrimp, finfish, penshell, and clams. There are two large fishing cooperatives (~ 80 members each), and currently, they have many small familiar aquaculture cooperatives, where families up to 10 members get permits to develop aquaculture from the state government; the requirements are to have an area willing to develop and have a family to work with the aquaculture, as the state government requires. Some members of Community B started their aquaculture projects back in 2000, while the majority started in 2014 following the oyster aquaculture policies implemented by the government (SEPADA 2022). Specifically, the governor of Baja California Sur promoted the transition to oyster aquaculture in the community by easily granting aquaculture permits to anyone who wanted one and providing assets (seeds, equipment, and materials) (CONAPESCA 2020; SEPADA 2022). The community has actively engaged in oyster aquaculture since its initial adoption, and this is still the case.

#### Honduras—Community C

Community C (Fig. 1) is predominantly made up of the Garifuna ethnic group, for whom fishing is not only a primary livelihood but also a deeply rooted cultural practice. These traditional fishers operate both in the open sea and the coastal lagoon adjacent to their village. The area is home to two fishing cooperatives that include a range of community members. Fishers use different types of nets and hook-and-line techniques to catch species like snappers, jacks, and snooks (Carbajal et al. 2017; Rivera and San Martin 2019). Over time, both the fishers and the wider community have observed a decline in fishery resources, which they attribute to climate change and the presence of outsiders fishing in the area without adhering to established regulations (Rivera and San Martin 2019). The fishing zones are subject to regulations enforced by the Honduran Fisheries Agency (DIGEPESCA). Additionally, an interinstitutional committee, comprising NGOs such as PROLANSATE, AMATELA, and the Coral Reef Alliance (CORAL), as well as government agencies like the Institute for Forest Conservation (ICF), plays a vital role in the management and enforcement of these regulations, ensuring sustainable practices in both the fisheries and the surrounding areas (Carbajal et al. 2017; Rivera and San Martin 2019). With the decline of wild-caught fisheries affecting both the environment and the livelihood of the community, CORAL, in collaboration with the committee, has engaged with local residents to introduce a native finfish aquaculture as a sustainable alternative. Through regular visits, they have provided education on the principles of aquaculture and its potential benefits, ensuring the community understands what it entails and how it can support their way of life.

#### Honduras—Community D

Community D (Fig. 1) is predominantly Garifuna and hosts the largest Garifuna population in the region, where fishing is a primary livelihood for many. The community relies heavily on open ocean fishing, primarily using hook-and-line techniques. There are two fishing cooperatives in the area, with a fish processing center managed by the cooperatives. These cooperatives include fishers, non-fishers, and women. The primary species caught are snappers, groupers, and jacks (Carbajal et al. 2017; Rivera and San Martin 2019). Similarly to Community C, over time, fishers and community members observed a noticeable decline in fishery resources, which they largely attribute to the effects of climate change and the presence of outside fishers who fail to comply with established regulations. As with Community C, the fishing areas are regulated by the Honduran Fishery Agency (DIGEPESCA). An interinstitutional committee, comprising non-governmental organizations like PROLANSATE, AMATELA, Coral Reef Alliance, and governmental agencies such as ICF (responsible for managing protected areas), assists in the management, regulation, and implementation of fishery practices in these areas (Carbajal et al. 2017; Rivera and San Martin 2019). Similarly to Community C, CORAL, in collaboration with other institutions, has engaged with this community to explore the potential for developing aquaculture of a native finfish.

### Collection and analysis of data

This study adopts a multistage mixed-methods approach, using exploratory mixed methods, integrating qualitative and quantitative data collection and analysis (Creswell and Clark 2007; Naku and Afrane 2013) during 2021–2024. The different participants were chosen based on those community members, fishers, and other stakeholders who have been involved in aquaculture, have experience working with it, and are also part of the fisheries, following purposive and snowball sampling approaches (Biernacki and Waldorf 1981; Etikan 2016). All interviews, ethnographic research, and surveys were conducted in Spanish. Due to the sensitive nature of the interviews and participant observations, the full dataset is not publicly available in order to protect participant confidentiality. However, the data have been fully anonymized (all identifying information removed) and are securely stored in encrypted, password-protected files in accordance with institutional data management protocols. Anonymized survey results and selected de-identified excerpts from interviews may be shared upon reasonable request. The lead author translated interview transcriptions, field notes, memos, and surveys into English to ensure accuracy and consistency in the analysis and interpretation of results. The analysis was done using Nvivo and Microsoft Word. Table 1 comprehensively summarizes the various research methods employed throughout the study. Each method is further detailed in the subsequent sections to illustrate its role in data collection and analysis.

**Table 1:** Summary of the different methods for the collection and analysis of data

Step	Part 1. Summer 2021—Communities A and B, Mexico	Part 1. Summer 2022—Communities A and B, Mexico	Part 2. Summer 2023—Communities A and B, Mexico	Part 2. Summer 2023—Communities C and D, Honduras	Part 2. Summer 2024—Communities C and D, Honduras
Data Collection	Direct participant observation Interviews (n = 13) (Supplementary material S1)	Direct participant observation Surveys (n = 44) (Supplementary material S2)	Focus groups (3,each with 5–8 participants) (Supplementary material S4)	Focus groups (3, each with 6–12 participants) (Supplementary material S4)	Direct participant observation Follow-up interviews (n = 13) (Supplementary material S5)
Analysis	Inductive grounded theory and thematic coding	Descriptive statistics, inductive grounded theory, and thematic coding	Inductive grounded theory and thematic coding	Inductive grounded theory and thematic coding	Inductive grounded theory and thematic coding
Justification of method	Captures nuanced, community-grounded insights from fishers with long-term experience in aquaculture, revealing values, concerns, and everyday practices often missed by structured methods	Enabled comparison across a broader group and provided statistical insights into community perceptions of aquaculture	Facilitated in-depth group discussions that surfaced the most important community-identified considerations for aquaculture implementation	Facilitated in-depth group discussions that helped surface key community-identified considerations for aquaculture implementation; particularly valuable in communities still in the planning phase, where semi-structured questions provided necessary guidance to explore expectations and concerns despite limited direct experience	Follow-up interviews in Honduras allowed deeper exploration of emerging systemic issues identified by communities, providing space to clarify and expand on concerns as aquaculture planning progressed

#### Part 1

In Part 1, qualitative data were gathered through ethnographic research and direct participant observation with detailed field notes (Supplementary Material S3) and interviews (2021–2023). These included thirteen interviews collected in 2021 (eight with fishers and community members involved in aquaculture, two with government officials, and three with individuals from NGOs or private companies promoting aquaculture in the area). Semi-structured interviews and participant observation were chosen to create space for participants to share their perspectives in their own terms (Bernard 2011). This approach enabled a deeper understanding of the social, cultural, and political dimensions of aquaculture implementation, allowing community members and other stakeholders to express experiences that might be overlooked in more structured or extractive methods. The field notes from ethnographic immersion and interviews were coded using an inductive grounded theory approach to thematic coding (Bernard 2011; Lawrence and Tar 2013).

The themes that emerged from the inductive grounded theory analysis aligned with core elements of the social–ecological systems (SES) framework, specifically, economic assets, governance, social organization, and culture and traditions (Partelow et al. 2018). To ensure clarity and relevance, we adopted a simplified version of the SES framework, focusing on these four components as identified by the communities themselves. While we acknowledge that SES frameworks typically encompass a broader range of interacting elements, this focused categorization allowed us to remain grounded in the lived experiences and priorities of community members engaged with aquaculture. This approach facilitated a deeper, context-specific interpretation of the results, revealing how aquaculture systems interact with existing fishery systems across diverse social and ecological dimensions (Ostrom 2009; Partelow et al. 2018, 2022).

For the quantitative part of Part 1, surveys with Likert scale questions (Supplementary Material S2) were developed and implemented, based on the qualitative data and resulting themes. The purpose of this was to understand the perceptions of community members of what they consider the most critical for the implementation of aquaculture, from what was discussed in the qualitative collection of data. Purposive and snowball sampling were used to target those specific community members and fishers who were actively participating in aquaculture and/or had participated in aquaculture in the past and had shared their experiences qualitatively. These surveys were administered to community members and fishers between June and July 2022 in Community A (n = 24) and Community B (n = 20).

#### Part 2

Part 2 in Mexico involved conducting three focus groups (Supplementary Material S4) (approximately one hour each) using semi-structured questions (Supplementary Material S4) with community members from Community A and B and local stakeholders (5–8 participants per focus group) in 2023. The goal was to further assess the key components identified in Part 1 and evaluate their relevance in the context of aquaculture implementation and potential expansion. These discussions provided insights into local priorities, challenges, and considerations, ensuring that community perspectives were integrated into aquaculture planning and decision-making. All the analysis was done using inductive grounded theory and thematic analysis.

For Honduras, Part 2 emerged as an opportunistic collaboration in which an NGO, working with local stakeholders, sought to implement aquaculture in two communities in Honduras (Fig. 1), but lacked a comprehensive understanding of the holistic requirements for a community-centered implementation, and wanted to have a participatory approach to evaluate the implementation of aquaculture with the communities, who are still in the planning process for such development. Seizing this opportunity, we showcased how the identified critical considerations from other communities’ experiences (Part 1) can inform participatory processes for the evaluation of the implementation of aquaculture in a proactive manner. As a result, the methodological approach integrated multiple epistemologies, prioritizing knowledge-sharing and participatory engagement over a strictly classical scientific framework (Charles et al. 2020). This opportunity allowed us to showcase how cross-community knowledge exchange, using previously identified key components and critical considerations, can inform context-specific participatory approaches for the implementation of aquaculture in small-scale fishery communities. To achieve this, we conducted two focus groups (Supplementary Material S4) (~ 1 h each) in 2023 with Communities C and D in Honduras (Fig. 1) and one focus group (~ 1 h) with local Honduran stakeholders in 2024 (with 6–12 participants each). Follow-up interviews (n = 13) were conducted in 2024 to deepen and expand upon the insights generated during the focus groups (Creswell and Clark 2007). Although the communities involved are in different countries, their experiences and socioeconomic characteristics provided valuable insights into the challenges and opportunities of small-scale fishing communities when aquaculture is implemented. While each country and community have unique social and economic characteristics, small-scale fishing communities share commonalities in their livelihoods, resource dependencies, and governance structures, allowing for meaningful comparisons. This comparative approach not only supports context-specific and participatory implementation but also contributes to broader discussions on how aquaculture can be adapted and scaled in fisheries-dependent communities.

## Results

Table 2 demonstrates the summary of the different representative results obtained across communities for the different components to be considered for the implementation of aquaculture.

**Table 2:** Summary of results per theme found in different communities.

Component	Community A—Mexico	Community B—Mexico	Community C—Honduras	Community D—Honduras
Culture and traditions	**Somewhat attached to traditions commitment to traditional knowledge** “T*hey are willing to learn more about aquaculture, but their cultural knowledge and attachment to their fishery customs weigh in a lot on the decisions they make regarding aquaculture”*	**Not attached to traditions but deep commitment to traditional knowledge** *“It is still important to have the traditional knowledge of fishing because when there is no market for aquaculture, we can still fish”*	**Importance of traditional knowledge, respect for traditions** *“All the youth from the community is leaving to go to the United States of America, we will reach a moment where all the fishery knowledge will be lost because the youth do not want to learn new alternatives, they just want to leave”*	**Importance of traditional knowledge, respect for traditions** *“Using native species for the aquaculture project”*
Economic assets	**Economic challenges for communities** *“Market is the most important, for the aquaculture products the market is different than from the fishery products”*	**Economic challenges for communities** *“With fishing, we are used to getting the money and spending it all at once, with aquaculture you have to learn to save it and invest, especially at the beginning of the project”*	**Economic challenges for communities, and impact of migration** *“We will use our backyards for the aquaculture”* *“It can affect these initiatives negatively, if we give assets and train a certain group of people for aquaculture, and they all migrate, it will negatively impact the project”*	**Economic challenges for communities** *“If we achieve the high production levels we aim for, we will need to sell in other cities”*
Governance	**Surveillance of aquaculture area is fundamental** *“You need to invest in the monitoring of the area, so they do not steal the harvest from the area”*	**Outside support** *“At the beginning, it is important that the government supports you”*	**New rules and agreements are needed** *“Clear internal agreements and rules must be established to manage the aquaculture project effectively because it is different from fishing”*	**Trainings are needed** *“The communities need different kinds of training for these aspects need to be considered before the implementation of an aquaculture project”*
Social organization	**Importance of including all family and women** *“It is good women are part of the oyster aquaculture now, the rest of the fishers want to go dive for scallop, and nobody wants to support the aquaculture because they do not like it”*	**Importance of including all family and women** *“When there was the big fishing cooperative doing aquaculture it was a mess, everyone wanted to be a leader, and everyone was fighting in the community. We learned that having small groups is better for working with aquaculture, it is more organized”*	**Smaller groups for aquaculture are needed** *“The way they currently organize the fishing activities is very different from what would be required for aquaculture operations, for aquaculture there will be smaller groups”*	**Smaller groups for aquaculture are needed** *“As fishermen, we tend to we are all leaders when we are fishing, and this sometimes generates conflict. For aquaculture we need to think there is one leader and work together”*

### Mexico

#### Mexico—Community A

**Fig. 2:**
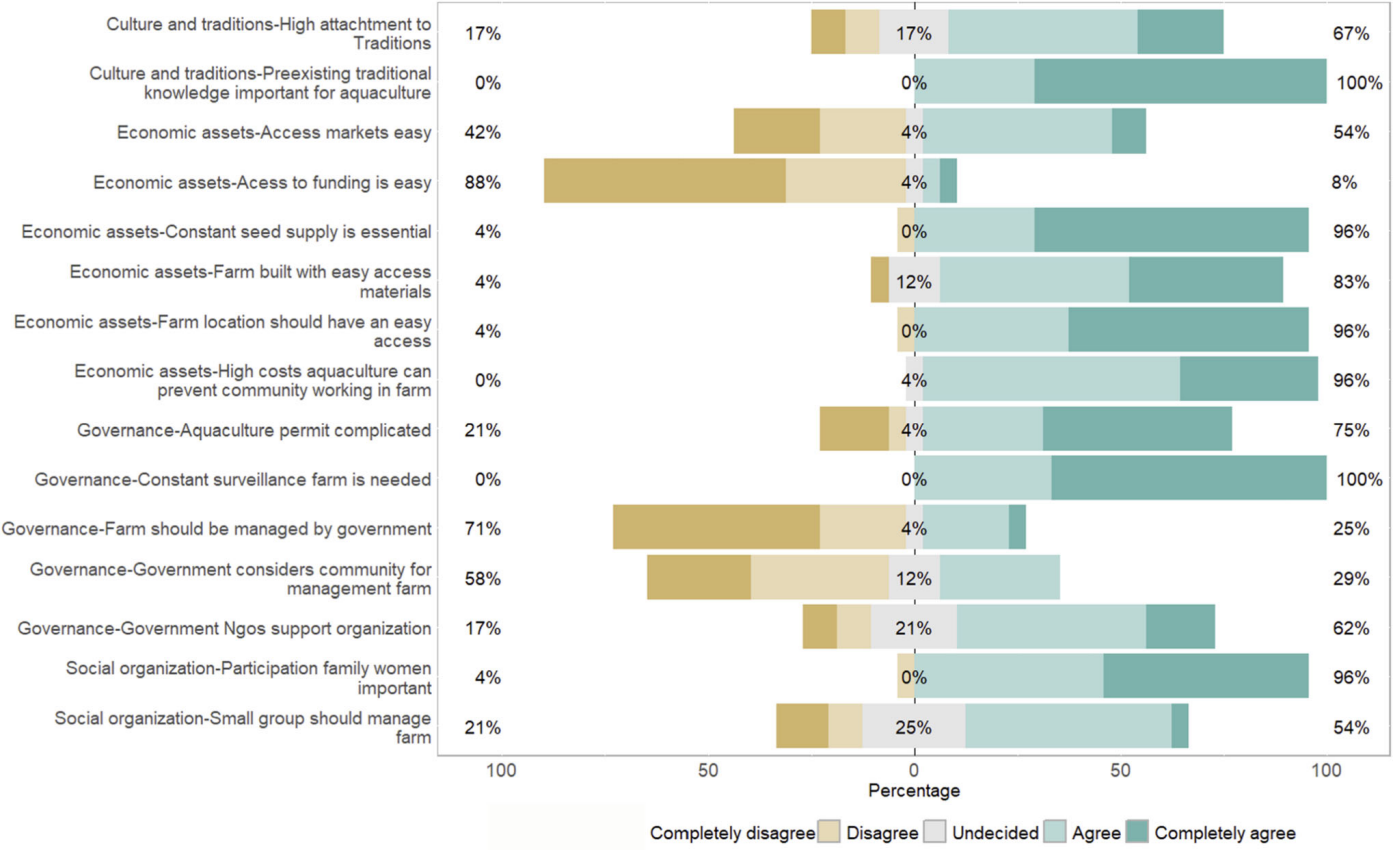
Community A perspectives on critical considerations for the implementation or expansion of aquaculture in their fishery system

Community A’s engagement with aquaculture provided valuable insights into critical considerations for its implementation (Fig. 2). Below, we present the community’s experiences, lessons learned, and the factors they consider important before expanding aquaculture or implementing new initiatives. It is essential to emphasize that this analysis reflects the community’s lived experiences and does not aim to provide a comprehensive evaluation of all aspects of the system relevant to aquaculture implementation.

#### Culture and traditions

Regarding their culture and traditions, 67% of surveyed community members agree that there is a strong attachment to traditions associated with the community and their livelihoods, as one of the fishers expressed, *“Diving is the only thing we know how to do*,” this needs to be considered when implementing aquaculture as it can impact the commitment of fishers to work with aquaculture. Furthermore, 100% of the surveyed community members agree there has to be a preexisting traditional knowledge such as a traditional understanding of the fishery and local environment—while equally valuing the ability to acquire new skills, both of which are essential for the effective functioning of the aquaculture farm (Fig. 2). As an employee from a local NGO expressed, *“They are willing to learn more about aquaculture, but their cultural knowledge and attachment to their fishery customs weigh in a lot on the decisions they make regarding aquaculture.”*

#### Economic assets

In terms of assets, 96% of surveyed community members agree that maintaining a constant seed supply is crucial for the system's success and that this is very hard to achieve (Fig. 2). Additionally, 96% acknowledge that the high startup costs of the aquaculture farm can discourage other community members from continuing in aquaculture or cause them to leave the project early, a local NGO employee expressed, *“They also need to see some economic benefits from the aquaculture when compared with the benefits they see with fishing”* (Fig. 2). Furthermore, 88% recognize the challenges of securing funding, emphasizing the need to account for this when sustaining the farm, as a fisher said, *“We need enough and constant seed, and funding to go on with the project, at the beginning you need a lot of funding to go on with it”* (Fig. 2). As a result, 83% of respondents report in some instances that they utilize easily accessible materials, such as plastic bottles and recycled bags found at home, to maintain the farm. Moreover, 96% have experienced the importance of locating the farm in an easily accessible area to ensure smooth operations. When it comes to product commercialization, they recognize that traditional fishing sales channels do not necessarily apply to aquaculture products, highlighting the need to establish markets before launching or expanding any aquaculture projects (45% express that establishing markets is hard, and 54% express it is easy), as a fisher expressed, *“Market is the most important, for the aquaculture products the market is different than from the fishery products”* (Fig. 2).

#### Governance

For governance, 75% of individuals highlighted the complexity of obtaining aquaculture permits, noting that the difficulty behind them can impact the ability of the community to work in aquaculture and must be addressed moving forward (Fig. 2). All participants agree that continuous surveillance and monitoring of the aquaculture farm and surrounding areas are vital to the project's success, as a fisher expressed, *“You need to invest in the monitoring of the area, so they [other community members] do not steal the harvest from the area.”* Also, 62% of the respondents expressed the importance of having some sort of support (i.e., funding, technical, training, etc.) from a non-governmental organization or government (i.e., implementation of aquaculture), especially in the first year of operation of the farm. Furthermore, 71% of the community members disagree that the government should manage the aquaculture farm, while 58% of them express that the government usually does not consider them when making decisions regarding the aquaculture farm and its operations. They consider that the community’s voice should be included more often in the implementation of aquaculture in the community, as an employee from an NGO said, *“There has to be clear written agreements and rules on how they will manage the aquaculture project, as it is different from how they manage their fishery”* (Fig. 2).

#### Social organization

Regarding the social organization, 54% of surveyed community members believe that the management of the aquaculture farm should be handled by a small, cohesive group—a contrast to the larger groups typically formed for fishing, as a fisher said, *“You cannot have a big group like with fishing, you need a small group so it is easier to work.”* Over time, the community understood the critical role of involving women in aquaculture operations, with 96% of those surveyed emphasizing the importance of their participation, as a fisher expressed, *“It is good women are part of the oyster aquaculture now, the rest of the fishers want to go dive for scallop, and nobody wants to support the aquaculture because they do not like it”* (Fig. 2).

#### Mexico—Community B

**Fig. 3:**
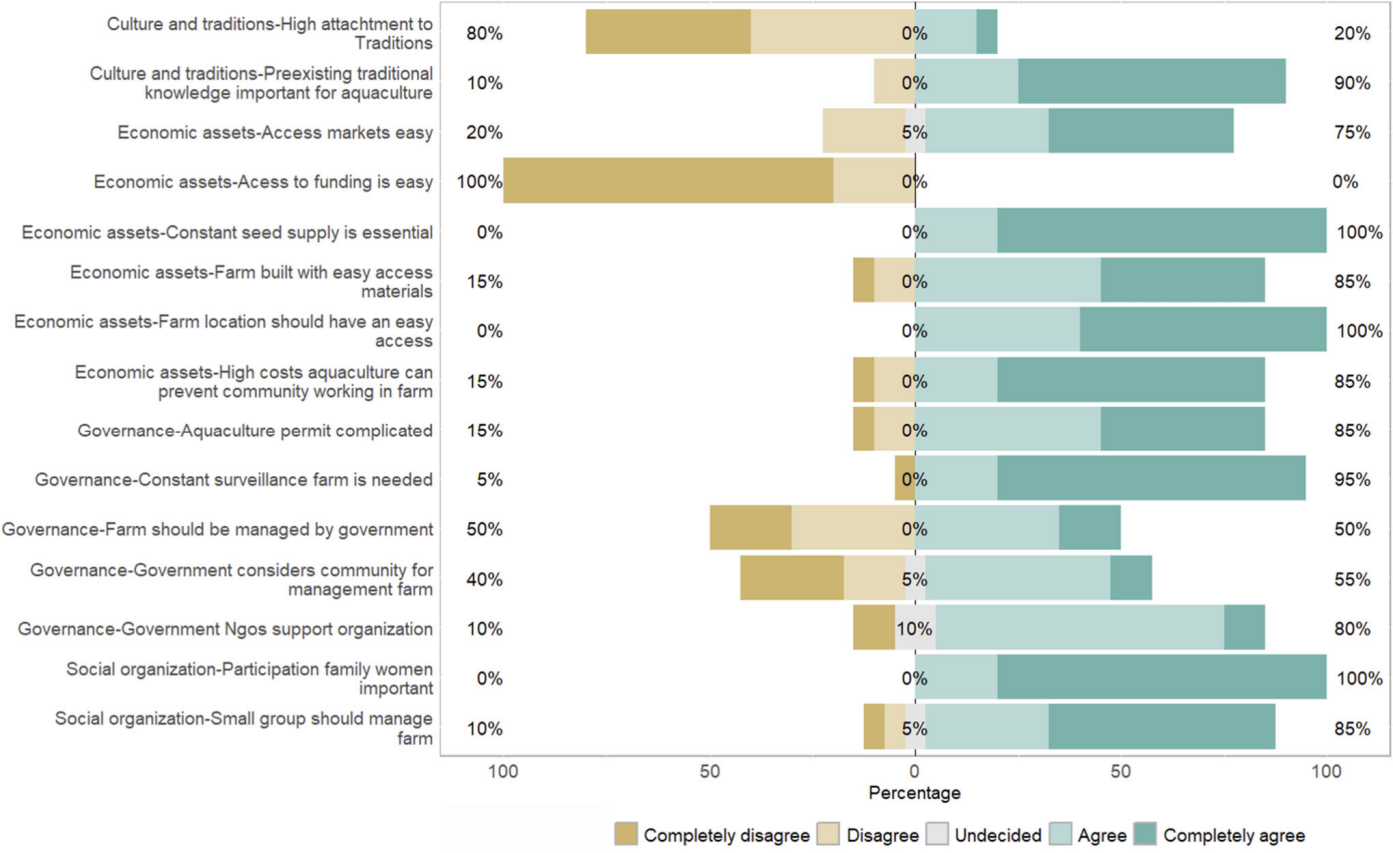
Community A perspectives on critical considerations for the implementation or expansion of aquaculture in their fishery system

Community B has gained different perspectives and insights from the implementation of aquaculture (Fig. 3).

#### Culture and traditions

For their culture and traditions, 80% of the individuals disagree that there is an attachment to the traditions of the community, which would impact the implementation of aquaculture. As a fisher said, *“We as fishers saw how bad the fisheries were, and it was too tiring and costly. So, we saw an opportunity with the aquaculture.”* However, as a fisher expressed, *“It is still important to have the traditional knowledge of fishing because when there is no market for aquaculture, we can still fish.”* Furthermore, 90% of respondents highlighted the importance of having preexisting knowledge and the capacity to learn new skills related to the aquaculture farm, a community member expressed, *“We have learned everything ourselves from mistakes”* (Fig. 3).

#### Economic assets

All surveyed community members (100%) agree that maintaining a steady and reliable seed supply is essential to the success of the aquaculture system, as they expressed, “*Having constant seed supply is essential for the aquaculture to work”* (Fig. 3). Moreover, all the individuals expressed that the high startup costs of the aquaculture farm affect the involvement of other community members from being part of the aquaculture project or making them leave the project early (Fig. 3). Furthermore, 100% of the surveyed individuals agree on how hard it has been to secure funding throughout their experience working with aquaculture. Additionally, the way fishers traditionally manage finances is different from aquaculture, as a fisher said, *“With fishing, we are used to getting the money and spending it all at once, with aquaculture you have to learn to save it and invest, especially at the beginning of the project”* (Fig. 3). All the community members surveyed agree that the location of the farm is very important, and it must be in a location that the community members have easy access to. For the materials used for the farm, 85% of the individuals have adapted to using materials they have easy access. Like Community A, the community members from Community B expressed the importance of establishing a market before the implementation or expansion of an aquaculture farm, 75% expressed it is currently easy to establish these markets.

#### Governance

Similarly to Community A, 80% of the individuals highlighted the importance of having support from an external organization, especially at the beginning of the operations of the farm, a community member expressed, *“At the beginning, it is important that the government supports you with money and materials…”* (Fig. 3). Additionally, 100% of the participants agree that the surveillance and monitoring of the aquaculture farm is very important for the success of the farm. Furthermore, 50% of individuals believe that the government should take responsibility for managing the aquaculture farm, 55% agree that the government involves them in decision-making processes related to the implementation and management of these farms, and the rest feel they should be more involved throughout the whole process (Fig. 3).

#### Social organization

85% of surveyed community members believe that management of aquaculture farms should be done by small, cohesive groups. In the words of one community member, *“When the aquaculture started, it was a mess because we were doing aquaculture with a big group like the big fishing cooperative, everyone wanted to be a leader, everyone was fighting in the community. But we had to learn on our own that for aquaculture, we work better in smaller groups. This way is more organized”*. Community B with time has modified its traditional male-dominated fishery system, to include women in their aquaculture operations, as they are critical for the implementation of aquaculture, as a fisher said,*“At the beginning, it was hard because of the machismo of our fisheries, but with time we realized how essential women are for the aquaculture”* (100% of the surveyed community members agree on this) (Fig. 3).

### Honduras

#### Honduras—Community C

##### Culture and traditions

Community members suggested using native species that hold high traditional and cultural value for aquaculture, which could also empower fishing communities. The older fishermen in the cooperative maintain a strong attachment to their fishing traditions and culture, yet they are open to exploring alternative livelihoods like aquaculture, a fisherman expressed, *“We will not stop fishing, if there is good weather, we would go out”*. Furthermore, community members possess a strong foundation of traditional knowledge and a deep connection to nature and local resources. However, community members expressed concern about the high migration rates and their culture and traditions, particularly among the youth, and how this could impact the implementation of aquaculture, a woman from the cooperative expressed*, “All the youth from the community is leaving to go to the United States of America, we will reach a moment where all the fishery knowledge will be lost because the youth do not want to learn new alternatives, they just want to leave”*.

#### Economic assets

For a future aquaculture project, like the one the NGO is interested in pursuing with the community, focus group participants (from the focus group with the community, and with the government and NGO participants) emphasized that while fishers and fishery cooperatives currently lack the necessary funding for such an initiative, they believe they could potentially secure financial support through government programs and NGO partnerships, however as a government employee expressed, *“There has to be an interest from the communities, if they don’t have any real interest in the aquaculture, the funding given will be lost.”* They recognize the need for essential equipment to support aquaculture operations, such as seed, feed, and an adequate location for the aquaculture farm, a fisherman expressed, *“We will use our backyards for the aquaculture.”* Community members also noted that they would need to gradually *“establish new markets for the aquaculture harvest”*, as the product would differ from what they typically sell from fishing, especially if the aquaculture operations grow with time. However, migration can threaten the viability of aquaculture initiatives, as a representative from an NGO said, *“It can affect these initiatives negatively, if we give assets and train a certain group of people for aquaculture, and they all migrate, it will negatively impact the project.”*

#### Governance

Participants recognized that there must be *“clear internal agreements and rules must be established to manage the aquaculture project effectively because it is different from fishing.”* A woman from the community expressed, *“If we do not write things clearly, then conflicts arise with the same community members”*. Moreover, they recognized the need to adjust the cooperative's legal structure, allowing them to commercialize aquaculture products, as the current legal framework of the fishing cooperative does not permit this. The participants suggest they would be in charge of managing the aquaculture farms, however, as a fisher expressed, “*We would work as allies with the government and NGO’s*.” They are also aware of the logistical difficulties in monitoring aquaculture farms, emphasizing that locating the farms close to their homes would be ideal for easier monitoring. They recognize the need for additional training to ensure the success of the aquaculture project, furthermore, as an NGO employee mentioned, “*The community members need to understand what aquaculture is, they know what fishing is, but they do not know how to operate or manage aquaculture.”* Specifically, they emphasized the importance of receiving training in project management and financial management, as the financial practices they use for fishing differ significantly from those required for aquaculture. Additionally, they acknowledged the necessity of technical training for operating an aquaculture farm.

#### Social organization

Key members of the community are migrating, which can impact the social organization. As a person working with a local NGO said, *“This community is very well organized, however, we are losing many leaders due to migration. We need to support them a lot, we need to continue supporting the organization and strengthening it.*” Community members emphasized the need to restructure the cooperative into smaller, more cohesive groups to manage the aquaculture projects. They noted that, *“The way they currently organize the fishing activities is very different from what would be required for aquaculture operations, for aquaculture there will be smaller groups”*. They also emphasize the importance of involving the community throughout the entire process of the aquaculture project, from its initial implementation to its ongoing management, ensuring that their voices and perspectives are integral to the project's success. Additionally, the women in the cooperative expressed a willingness to participate in the aquaculture project, as a fisher said, *“All the families should be involved in the project”*; similarly, a government official also said, *“In this type of project we can include all the family, even the women, which is different than the traditional fishing of the community”*.

#### Honduras—Community D

Similarly to Community C, community members, and stakeholders, shared their insights on the necessary changes and key factors for successful aquaculture implementation.

##### Culture and traditions

The members of Community D expressed the importance of *“using native species for the aquaculture project”*, as it will make them *“feel empowered.”* Furthermore, the fishers expressed how they have a high traditional knowledge about their surroundings and fishery resources. Similarly to community C, the older fishers in the cooperative and community maintain a deep attachment to their fishing traditions, yet they are open to participating in the aquaculture project as well, as the director of an NGO expressed, *“This community can work on different economic alternatives, they need it.”* The participants acknowledge the importance of involving families and integrating the aquaculture initiative into their children's education, to preserve and pass down knowledge that is currently being lost due to migration, as one NGO employee noted, *“In this community, we are losing the knowledge of fishing, the youth are leaving the community, and this worries the fishery groups of the community.”*

#### Economic assets

Focus group participants emphasized that the project's funding should combine community and external sources. They noted that when funding was solely external in past initiatives, *“The community showed less commitment to the project.”* Community D recognizes that if aquaculture is wanted, it needs to expand its market beyond the local community eventually. As one fisher noted, *“If we achieve the high production levels we aim for, we will need to sell in other cities.”*

#### Governance

Community D expressed concerns about the impact of youth migration on the management of the aquaculture project, as their fishery cooperatives are affected, and they think that it would also impact the structure to manage the aquaculture project. Additionally, they also state how they should create new rules to manage the farm, *“We need to create new rules to avoid people not working and creating conflict with the community”*. However, the community members agree that they need more training for the implementation of aquaculture, as a fisher expressed, “*The more training the better it is for us.”* Furthermore, government officials and NGO employees all agreed that, *“The communities need different kinds of training for these aspects need to be considered before the implementation of an aquaculture project.”*

#### Social organization

Similarly to Community C, this community expressed the importance of including women in the entire aquaculture project, a fisherman expressed, *“They are better at making decisions than us.”* The community recognizes they are organized; however, they noted that they need to learn how to work in more cohesive groups which they recognize is needed for aquaculture, a fisherman expressed, *“As fishermen, we tend to we are all leaders when we are fishing, and this sometimes generates conflict. For aquaculture we need to think there is one leader and work together.”* Similarly to Community C, this community expressed it is losing its leaders due to migration, and this must be considered before the implementation of an aquaculture project.

## Discussion

The integration of aquaculture into coastal small-scale fishery communities is gaining momentum, particularly in less developed countries across the Global South, driven by Blue Economy narratives as well as other development frameworks focused on food security, poverty alleviation, and rural livelihoods (Brugere et al. 2021; Campbell et al. 2021; Farmery et al. 2021). However, many current efforts rely on top-down over-simplistic, technocratic approaches that emphasize economic and technical aspects (Barrett et al. 2002; Krause et al. 2020; Farmery et al. 2021; Funk et al. 2022; Brugere et al. 2023). These approaches tend to overlook the different components critical for the implementation of aquaculture (according to communities’ experiences) in these systems (besides economic and technical), and the interactions between aquaculture and the existing dimensions of fishery systems, treating aquaculture as a separate sector rather than one that both influences and is shaped by the social–ecological dynamics of the existing fishery system (Mansfield et al. 2024). Our findings highlight the urgent need to evolve top-down approaches by meaningfully incorporating the priorities and lived experiences of fishing communities. Rather than seeking to replace centralized decision-making, this research proposes a methodology to make such approaches more responsive, inclusive, and grounded in local realities. Our methods provide a pathway for developing community-defined priorities and contextual considerations, offering practical tools for embedding participatory and cross-scalar inputs into existing decision-making processes.

### Cultural and Social considerations in aquaculture development

All communities highlighted the importance of respecting cultural traditions and traditional knowledge when introducing aquaculture in small-scale fishery communities. Neglecting these traditions can lead to significant trade-offs, including the loss of traditional knowledge, decreased resilience, and threats to livelihoods (Murray et al. 2005; Pitchon 2015; Campbell et al. 2021; Baker-Médard and Kroger 2023; Mansfield et al. 2024). Ensuring cultural alignment between aquaculture practices and community traditions can minimize these potential trade-offs and strengthen community engagement with the aquaculture project (Kluger et al. 2019; Stoll et al. 2019). For example, participants from Community B acknowledged that while their attachment to traditional fisheries is not a barrier to aquaculture implementation, traditional knowledge remains essential for building resilience within the system (Galappaththi et al. 2021; Roscher et al. 2022). They emphasized that they cannot rely solely on aquaculture and that maintaining traditional practices strengthens their ability to adapt and sustain their livelihoods, which is important to build resilience for vulnerable coastal communities (Roscher et al. 2022). Communities in Honduras also emphasized the importance of using culturally significant species in aquaculture to empower local communities and honor their traditions; this can also lead to more motivation for fishers to work in aquaculture as seen in the sea cucumber aquaculture in Palau (Ferguson, n.d.). Ignoring these cultural aspects can create issues of justice and equity, such as the displacement or elimination of traditional fishing livelihoods (Barrett et al. 2002; Campbell et al. 2021; Mansfield et al. 2024).

### Economic challenges

Economic feasibility is another critical concern, particularly during the early stages of aquaculture implementation, when farms may not yield immediate returns (Belton et al. 2018; Brugere et al. 2021). This is particularly important as Community B expressed, fishers and fishing communities typically operate on immediate earnings, where saving and investing are not a priority (LS, personal observation, 2022). However, aquaculture requires financial planning, as income is delayed until harvest, adapting to this shift is crucial for aquaculture adoption (Albers et al. 2021a). All communities highlighted the need for consistent seed supply, accessible financial support (i.e., loans, subsidies, tax incentives), adequate location of aquaculture farms, and measures to prevent financial strain (Costa-Pierce 2010; Gerhardinger et al. 2023; Fong et al. 2024). Additionally, all communities recognized the importance of establishing markets before aquaculture implementation, as market dynamics for aquaculture products often differ from those of traditional fisheries, and if not considered could create market competition with fishery products sold at traditional fishery markets (Asche et al. 2005; Kluger et al. 2019; Damonte et al. 2023). However, a sole focus on economic assets without integrating governance, social organization, and cultural factors can destabilize existing fishery systems (D’Anna and Murray 2015; Campbell et al. 2021; Brugere et al. 2023; Mansfield et al. 2024). If economic and technical priorities overshadow other social considerations (i.e., governance, culture, etc.), both the aquaculture farm and the fishery system might be affected (Mansfield et al. 2024). For example, in Madagascar, the focus on the increase of sea cucumber mariculture affected the social relations that existed in the small-scale fishery (Baker-Médard and Kroger 2023).

### Social organization

For the social organization component, all communities recognized the importance of restructuring large fishing groups into smaller, more cohesive units before implementing aquaculture. Smaller groups can foster trust (Ateweberhan et al. 2018), enhance collaboration, and provide opportunities to address freeriding issues, which can lead to positive synergies for both aquaculture and fisheries (Pitchon 2015; Sepúlveda et al. 2019; Albers et al. 2021b; Mansfield et al. 2024). Without such restructuring, aquaculture might impact the social dynamics of the existing fishing community (Mansfield et al. 2024), as communities A and B experienced with their aquaculture projects (i.e., increased fights within the community, and loss of trust among each other). Additionally, all communities emphasized the need to include families and women in aquaculture activities (von Essen et al. 2013; Hair et al. 2019; Brugere et al. 2023 ). Incorporating women not only challenges them but also enhances the traditionally male-dominated fishery system (Baker-Médard and Kroger 2023). This apparent contradiction (traditional male fishery-dominated systems vs aquaculture) reveals a shift in collective thinking, although existing norms may limit women’s participation; communities increasingly view aquaculture as an opportunity to redefine roles and promote more equitable household and community engagement. However, integrating women into aquaculture activities must be approached with care, as it is not a straightforward process. If not managed thoughtfully, it can disrupt existing power dynamics, reinforce inequalities, or create new risks (Fröcklin et al. 2012; Baker-Médard and Kroger 2023; Knott et al. 2024).

### Governance and institutional support

The results highlight that aquaculture governance and management differ significantly from traditional fisheries, offering opportunities for collaboration among communities, governments, and organizations such as NGOs, with approaches varying based on local contexts (Krause et al. 2020; Partelow, 2023; Partelow et al. 2023). Community A exemplifies a setting where collaboration between local NGOs and community members could support aquaculture development, especially in understanding permitting structures for aquaculture operations, showcasing an example where the inclusion of external agents can be beneficial for the community. In Community B, while surveyed members acknowledged government support for aquaculture implementation, some preferred a community-led management approach, allowing them to establish their own regulations and governance structures with government support, as there might be concerns that a government-led approach might overlook community voices (Partelow et al. 2022).

Similarly, in the Honduran communities, participants expressed a strong desire to lead aquaculture management while engaging in partnerships with the government and NGOs. Moreover, participants in these communities suggest clear, written agreements and guidelines on aquaculture management to prevent potential negative effects on the existing fishery system, such as conflicts over space with fishers, which could negatively impact existing fisheries, and create social and ecological issues (Pitchon 2015; Mansfield et al. 2024), and mitigate other unintended consequences, external agents such as governments and NGO’s can help communities lead these efforts (Partelow et al. 2022). Communities A and B agreed that the monitoring and surveillance of aquaculture farms is critical, particularly when farms are located near fishing areas. Unregulated aquaculture farms may lead to conflicts over space with fishers, which could negatively impact existing fisheries (as expressed by Community A), and create social and ecological issues (Pitchon 2015; Mansfield et al. 2024). While formal surveillance of fishing areas and aquaculture farms may be burdensome or unsustainable for governments and NGOs, these actors can play a critical role in supporting communities to develop and lead their own informal monitoring and enforcement systems. By facilitating the creation of locally legitimate rules and community-based surveillance mechanisms, external actors can help strengthen compliance and governance from within (Gomez-Andujar et al. n.d.; Sierra Castillo et al. 2024).

Communities C and D also emphasized the importance of training in financial management, project management, and team strengthening, in addition to technical aquaculture training (especially before the implementation of aquaculture). These skills can help reduce the costs of implementing an aquaculture project, which might otherwise increase without such considerations (Partelow et al. 2022; Mansfield et al. 2024). Furthermore, these communities highlighted a unique challenge, the need to modify the legal structure of their fishery cooperatives to commercialize aquaculture products, a regulatory hurdle specific to the area.

### Participatory approaches and knowledge exchange

Case studies from Communities C and D highlight the importance of participatory approaches (Charles et al. 2020) in implementing aquaculture within fishing communities. By leveraging the experiences of communities with established aquaculture expertise from other communities (Communities A and B), knowledge-sharing initiatives can help simplify complex systems and proactively identify key considerations for implementation, which can be fundamental for community-centered approaches to aquaculture. These insights from experienced communities can help mitigate the risk of trial-and-error learning (Charles et al. 2020; Hakkarainen et al. 2020). Instead of repeating common mistakes, communities can build on proven strategies, leading to more efficient and sustainable adoption of aquaculture practices. These collaborations among communities, NGOs, and governments (or other external agents) ensure that aquaculture is tailored to the unique socioeconomic context of each community, fostering more informed and sustainable decision-making (Brugere et al. 2023; Mansfield et al. 2024).

For instance, Honduran communities expressed concerns about how migration is disrupting their cultural knowledge, governance, and social organization structures, leading to the loss of community leaders and traditional knowledge. This affects not only the fisheries (Asiedu et al. 2023; Griffith, 2020; Islam and Herbeck, 2013), but also the potential development of aquaculture. Overlooking these systemic challenges can result in costly failures, particularly when financial investments in aquaculture projects are allocated to individuals who later migrate, as expressed by NGO employees in Honduras. Addressing these issues proactively can help mitigate such risks by integrating entire families into aquaculture initiatives and fostering new leadership within communities. Furthermore, acknowledging these challenges benefits both aquaculture and the broader fishery system. Strengthening leadership structure, incorporating whole families, and creating governance structures for the implementation of aquaculture, while preserving traditional knowledge can create positive synergies that will help reduce the negative impacts of migration on a potential aquaculture initiative while strengthening the fishery system that is being impacted by migration (as reported by Communities C and D).

### Rethinking aquaculture implementation

Our findings challenge linear, one-size-fits-all, simplistic technocratic approaches to the implementation of aquaculture in small-scale fishing communities. Instead, we advocate for iterative approaches that prioritize communities’ voices and experiences in decision-making, address systemic inequities, and recognize the interactions between fisheries and aquaculture systems (and between their components). These systems inherently interact; changes in one inevitably impact the other, just as their components are interwoven, continuously influencing and shaping each other (Bennett et al. 2019; J. L. Blythe et al. 2021; Farmery et al. 2021; Roscher et al. 2022; Mansfield et al. 2024).

While our case studies provide valuable insights, they focus on the social and economic components essential for aquaculture implementation; the environmental, ecological, and production aspects are equally important. Future research should explore the ecological, environmental, and production dimensions of these system interactions with the social components we describe in this study, to ensure a more holistic understanding. The collaboration between NGO’s, governments, and communities can be fundamental to achieving this (Mansfield et al. 2024). Longitudinal studies are needed to examine the evolving interactions between aquaculture and fisheries over time. Empirical data can help uncover the intricate connections within social–ecological systems, shedding light on how these relationships vary across different contexts, and how different components might be critical in different communities (Brugere et al. 2023; Mansfield et al. 2024).

This broader perspective will be crucial in identifying the key factors that influence aquaculture integration in small-scale fishing communities, particularly in regions with distinct socioeconomic characteristics, ensuring that implementation strategies are both effective and contextually appropriate. Aquaculture presents significant opportunities, including economic benefits, livelihood diversification, and gender inclusion (Campbell et al. 2021; Mansfield et al. 2024). However, realizing these benefits requires thoughtful, inclusive planning that addresses systemic challenges, recognizes the existing social–ecological dynamics of the fishery system and its interactions with aquaculture, and places communities at the core of the process. Failing to do so threatens the intended benefits of aquaculture, potentially leading to unintended consequences that may harm rather than support the small-scale fishery communities where it is implemented (D’Anna and Murray 2015; Mansfield et al. 2024).

## Supplementary Information

Below is the link to the electronic supplementary material.

Supplementary file1 (PDF 380 kb)
