# Supplementary material for: Community-centered approaches to aquaculture in small-scale fisheries
Source: Ambio. 2025 Dec 18;55(6):1304–21. doi: 10.1007/s13280-025-02302-w (PMC13125479; doi:10.1007/s13280-025-02302-w)
Supplement: Supplementary file 1 — Supplementary file1 (PDF 380 kb) [file 13280_2025_2302_MOESM1_ESM.pdf]

Ambio

Supporting Materials

This supplementary information has not been peer reviewed.

Community-Centered Approaches to Aquaculture in Small-Scale Fisheries

Contents of this file

Supplementary material S1 to S4, Figure S1

Supplementary material

S1. Interview questions Mexico communities, and stakeholders (Summer 2021)

Thank you for agreeing to participate in this interview. Your responses will remain completely anonymous. If there's any question you're uncomfortable answering, just let me know and we can skip it. You're also free to stop the survey at any time. I will be recording this interview for purposes of analysis, if at any time you feel uncomfortable with it let me know, and we can stop the recording.

1. Tell me about your experience as a fisher working in aquaculture?
2. What is needed for the aquaculture to be successful in your experience?

S2. Survey questions developed from interview analysis, Mexico communities (Summer 2022)

Thank you for agreeing to participate in this survey. Your responses will remain completely anonymous. If there's any question you're uncomfortable answering, just let me know and we can skip it. You're also free to stop the survey at any time.

Prompt. Please tell me how much you agree or disagree with the following statements

1. Having constant access to seeds is fundamental for the aquaculture
2. High costs of aquaculture make communities not work in aquaculture
3. The aquaculture farm has to be in an accessible area for the community
4. It is easy to sell aquaculture products/ access to markets is easy
5. Aquaculture farms should be built with materials the community has access to
6. Is easy to get funding for aquaculture
7. Women and families should be involved in aquaculture
8. Aquaculture permits are complicated
9. Customs and traditions associated with the fishery/ community affect aquaculture
10. Previous traditional knowledge is important for the functioning of aquaculture farm
11. The group in charge of aquaculture should be small <10 people
12. There has to be constant surveillance on the farm
13. NGOs and government support aquaculture
14. The aquaculture farm should be managed and controlled by rules made by the government/external agent

15. The government/ external agent takes into consideration communities' voice and knowledge for the management of aquaculture

S3. To complement survey and interview data, I conducted direct participant observation during field visits, actively engaging in the daily livelihood activities of both male and female community members. These activities included fishing, processing, and community meetings. Each interaction was documented through detailed field notes, recorded in a field journal. Observational data were analyzed using inductive grounded theory, applying thematic coding to identify recurring patterns and meanings. These themes were integrated into the overall analysis, helping to refine survey instruments and enrich interview protocols with context-specific language and concepts. Observation guidelines were grounded in everyday livelihood interactions. Criteria for recording and systematizing observations included date, activity, setting, actors involved, and reflective notes, which were later coded using NVivo software and Microsoft Word.

Entry into communities was facilitated by local leaders, cooperative representatives, and prior acquaintances made through earlier work, and collaborations with fishing cooperatives, community members, and NGO's in Honduras and Mexico. Prior to engaging in participant observation, interviewing or surveying participicnants verbal informed consent was sought from both community leaders and individual participants. An actor map was developed through early interviews and observations, identifying key groups and individuals within the fisheries and aquaculture systems, including fishers, and community leaders. Fieldwork concluded with feedback sessions in each community, where I presented preliminary findings and engaged in discussion with participants. To support ongoing knowledge sharing, pamphlets summarizing key insights were distributed to community members.

The first stage of the research (Part 1) focused on the Mexico sites, where qualitative data was gathered through ethnographic fieldwork, participant observation, and semi-structured interviews conducted between 2021 and 2023. These methods created space for community members and other stakeholders to openly share their lived experiences and perspectives on aquaculture development within their small-scale fishery contexts, adding depth, nuance, and grounded context to the analysis.

In contrast, the Honduras case studies were incorporated later in collaboration with the Coral Reef Alliance (CORAL) and Garifuna community organizations. While similarly rooted in

community-engaged approaches, the first stage of qualitative data collection was not carried out in Honduras because the communities were still in the planning phase of integrating aquaculture into their fisheries. As such, they had limited experience or direct knowledge to reflect on implementation outcomes. Instead, semi-structured focus groups were used to provide a flexible yet guided format that supported participants in discussing their expectations, concerns, and early impressions of aquaculture, helping to surface locally meaningful insights during this formative stage.

S4. Focus group questions Mexico and Honduras, communities, and stakeholders (Summer 2023)

Thank you for agreeing to participate in this interview. Your responses will remain completely anonymous. If there's any question you're uncomfortable answering, just let me know and we can skip it. You're also free to stop the survey at any time. I will be recording this interview for purposes of analysis, if at any time you feel uncomfortable with it let me know, and we can stop the recording.

1. What would you change if you had to do aquaculture from the start?
2. General characteristics of their fishery system
    a. What do you fish and sell?
    b. What are your earnings when you go to fish?
    c. What management is available for the fishery?
    d. How is the community economically?
3. Characteristics of the fishery system per previously identified components
    a. Economic assets
        i. Does the community have access to funding, loans, etc.?
    b. Social organization
        i. Is the community organized and has leaders? Women and families involved?
        ii. The community has norms and values?
    c. Culture and traditions
        i. The community has a high attachment to their culture and customs associated with the fishery?
        ii. The community has a traditional knowledge?

   d. Governance
      i. How is the fishery managed? Cooperatives? Fishery associations?
      ii. Is the community involved in the management of the fishery? Who is involved?
      iii. What rules are there?
4. Characteristics of the aquaculture system/what is needed for aquaculture, per previously identified components
   a. Economic assets
      i. What is needed in terms of equipment, materials, funding, and technical aspects for aquaculture?
      ii. What about markets?
   b. Social organization
      i. How does the organization have to change for aquaculture? (i.e., make smaller groups, why?)
      ii. Does the relationship between community members have to be changed?
      iii. Include women and families?
   c. Culture and traditions
      i. If there is a high attachment to cultures and traditions of fishery, what needs to be considered for the community to work in aquaculture? How can we include it in the aquaculture?
      ii. Is the community willing to reduce fishing to work in aquaculture?
      iii. Besides traditional knowledge, what other knowledge is needed for aquaculture? How to consider traditional knowledge?
   d. Governance
      i. What rules should exist to manage aquaculture?
      ii. Who would be in charge of those rules and management of aquaculture? How is it different from fishery?
      iii. What trainings does the community need for aquaculture?
      iv. Where should farms be located? Who should monitor farms?

S4. Follow up interviews with Honduran communities and stakeholders (Summer 2024)

Prompt. If we recall the components discussed for the implementation of aquaculture (if they do not remember, discuss them again with them), I want to ask follow-up questions

a. What challenges do you anticipate happening with aquaculture and its components, and its interactions with the fishing community? Migration issues affecting components?
b. How do you see overcoming these challenges?

Supplementary figures

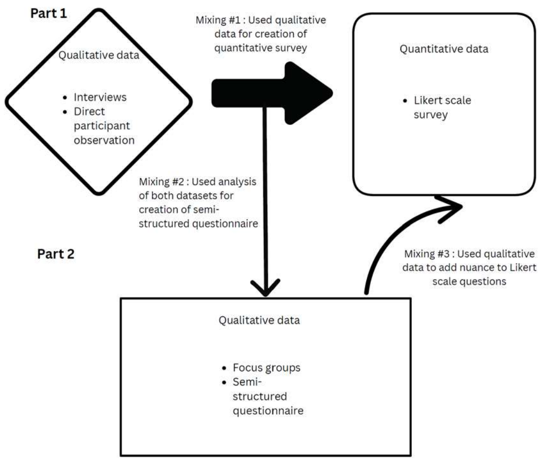

Figure S1. Different stages for the multi stage mixed methods, description of each stage, and its interactions
